# Meteorological fluctuations define long-term crop yield patterns in conventional and organic production systems

**DOI:** 10.1038/s41598-017-00775-8

**Published:** 2017-04-06

**Authors:** John R. Teasdale, Michel A. Cavigelli

**Affiliations:** Sustainable Agricultural Systems Laboratory, Agricultural Research Service, United States Department of Agriculture, 10300 Baltimore Avenue, Beltsville, Maryland 20705 United States

## Abstract

Variability in meteorological patterns presents significant challenges to crop production consistency and yield stability. Meteorological influences on corn and soybean grain yields were analyzed over an 18-year period at a long-term experiment in Beltsville, Maryland, U.S.A., comparing conventional and organic management systems. Precipitation and temperature variables explained much of the yield variability, with precipitation and heat stress during the late vegetative and early reproductive phases of crop growth accounting for the majority of yield variability in all crops and management systems. Crop yields under conventional and organic management followed similar periodic patterns, but yields were 31% and 20% lower in organic than conventional corn and soybean, respectively. The efficiency of grain yield per unit precipitation was higher under conventional than organic management, highlighting the importance of crop management for optimizing production in response to meteorological variability. Periodic yield and precipitation patterns did not consistently align with global meteorological cycles such as the El Niño Southern Oscillation.

## Introduction

Year-to-year variability in crop yield presents a challenging obstacle to achieving global food security. In a recent analysis of 13,500 local political units worldwide, 32 to 39% of global crop yield variability could be explained by climate variability^1^, amounting to potential fluctuations of 22, 9, 3, and 2 million tons in global corn, wheat, rice, and soybean production, respectively. Drought stress is often the dominant factor responsible for yield reductions^2^, and crop sensitivity to drought stress has increased over the past two decades^3^. Heat stress can interact with drought stress by increasing vapor pressure deficits and accentuating the inability of crops to access adequate soil moisture^4, 5^. Against this backdrop of climatic challenges to crop production stability are issues of food system sustainability and the potential degradation of essential environmental resources^6, 7^. Organic agriculture has been proposed as an alternative to conventional production practices that can improve the sustainability of current food production systems^8^.

Several recent meta-analyses of conventional versus organic farming have found that organic farming provides many benefits over conventional farming in environmental protection, improved biodiversity, higher food nutritional value, and positive economic returns. A summary of 71 publications found that European organic farms had significantly higher soil organic matter content and lower nitrogen leaching and nitrous oxide emissions per unit of field area, however, nitrogen leaching and nitrous oxide emissions per product unit were higher from organic systems^9^. This report also summarized 38 publications on biodiversity and found that most studies demonstrated higher species richness and abundance on organic farms. Another meta-analysis summarized 343 publications and demonstrated that concentrations of antioxidants were 19 to 69% higher in organic crops/foods, but the occurrence of pesticide residues was four times higher on conventional crops^10^. There was a positive difference in net returns per field area for organic versus conventional production of corn and soybean grown in the US, primarily because of price premiums paid for organic crops^11^. On the other hand, crop yield is often limiting on organic farms. A summary of 66 reports of 316 organic-to-conventional yield comparisons showed that, on average, yields of organically-produced crops were 25% lower than yields of conventionally-produced crops^12^. In a subsequent meta-analysis with upgraded analytical techniques, organic yields were shown to be 19% lower than conventional yields overall, but multi-cropping and crop rotations substantially reduced that yield gap to 9% and 8%, respectively, when these methods were applied in only organic systems^13^. Several reports suggest that ideal cropping systems should include a combination of high yield potential and low negative environmental impacts, drawing on the most sustainable techniques from both organic and conventional systems^8, 9, 12^.

Results from a long-term agroecological research project in Beltsville, Maryland, the Farming Systems Project (FSP), have confirmed many of the findings outlined above. Crop yields were higher in conventional than organic systems, but organic systems had higher soil carbon mass, nitrogen mineralization potential, biodiversity, and net economic performance, along with lower net greenhouse gas emissions than conventional systems^14–18^. Among organic systems compared at the FSP, longer, more diverse rotations significantly increased soil particulate organic carbon and nitrogen fractions and yield, while reducing economic risk, weed populations, corn yield loss to weeds, and soil erosion potential^15, 16, 18–20^.

One advantage of long-term agricultural research is the potential to determine long-term trends in cropping system performance as the systems mature^21^. The southeastern and mid-Atlantic coastal plain, where the FSP resides, is an area with higher than average variability in corn and soybean yields compared to the “breadbasket” regions of the world, and an area where precipitation is the primary contributor to variability^1^. This constraint on production provides a unique challenge to the design and functionality of mid-Atlantic cropping systems. In this paper, we focus on the inter-annual fluctuations in climate-driven crop yields across the 18-year span of the FSP experiment within conventional and organic management systems. Our objectives were to i) identify the factors most influential in determining inter-annual corn and soybean yield variations, ii) determine the comparative response of conventional and organic management systems over this time period, and iii) explore relationships among local periodic yield and precipitation patterns and global El Niño Southern Oscillation patterns.

## Results and Discussion

### Factors influencing crop yield

Variance decomposition of FSP yield data revealed that 74% of the variation in corn data and 68% of the variation in soybean data was explained by year-to-year variability. An additional 16% of variance for each crop was attributed to management group (conventional versus organic), whereas cropping system within group (no-till versus chisel-till within conventional management and two-year versus three-year versus six-year crop rotations within organic management) as well as field block accounted for less than 1% of variance each. Supplementary Figs S1 and S2 show the similar patterns of yield response across years among systems within conventional and organic management groups. Consequently, this paper focuses on the two factors that influenced yield variability most strongly, namely, year-to-year climatic factors and conventional versus organic management.

Several meteorological and management variables were identified that explained a large portion of the inter-annual variability of yield data in this experiment. Optimum multiple regression models explained from 72 to 87% of the variability in corn and soybean yields (Table 1). Late season precipitation had the highest correlation with yield (correlation coefficients ranging from 0.70 to 0.80) and was the first variable entered into multiple regression expressions (standardized coefficients ranging from 0.50 to 0.62), suggesting this was the most important factor that explained yield variability across years at this site. Early season precipitation also explained a significant portion of yield variability in conventional corn and soybean and organic corn, but not organic soybean.

**Table 1:** Correlation and standardized multiple regression coefficients for crop yields with climatic and management variables.

	Correlation coefficient^a^				Standardized multiple regression coefficient^a^			
	Corn		Soybean		Corn		Soybean	
Variable	Conventional	Organic	Conventional	Organic	Conventional	Organic	Conventional	Organic
Late precipitation^b^	0.80	0.70	0.71	0.79	0.54	0.50	0.54	0.62
Early precipitation^b^	0.53	0.41	0.68	NS	0.24	0.22	0.50	NS
Heat stress units^b,c^	−0.77	−0.75	−0.54	−0.48	−0.39	−0.38	NS	−0.21
Weed cover	NS	−0.31	NS	−0.45	NS	−0.21	NS	−0.28
Preplant tillage	NS	—	NS	—	NS	—	NS	—
Rotation	—	NS	—	NS	—	NS	—	NS
n	123	202	134	191	123	202	131	191
Multiple regression R^2^	—	—	—	—	0.87	0.79	0.73	0.72

^a^Correlation coefficients are significant at P < 0.01. Multiple regression coefficients are shown for factors that were entered and retained in the model according to a stepwise selection procedure at P < 0.01. NS designates coefficients that were not significant. A dash indicates that the factor was not included in that analysis. ^b^Precipitation and heat stress periods are defined in Supplemental Table S1. ^c^Heat stress units are the accumulation of daily maximum temperature above 30 °C.

Temperature variables were highly correlated with each other, with correlations between heat stress units and average temperature during the late critical period ranging from 0.84 to 0.91. Since heat stress had a higher correlation with yield than average temperature variables, and is considered a critical factor determining crop yield^4, 5^, this parameter was chosen as the most explanatory temperature factor for inclusion in multiple regression analyses. Heat stress was highly correlated with corn yield, and was second in explanatory power to late precipitation in the multiple regression analysis of corn yield (Table 1). Heat stress had a lower correlation with soybean than with corn yield, and had relatively little influence on soybean yield in the multiple regression analyses.

**Figure 1:**
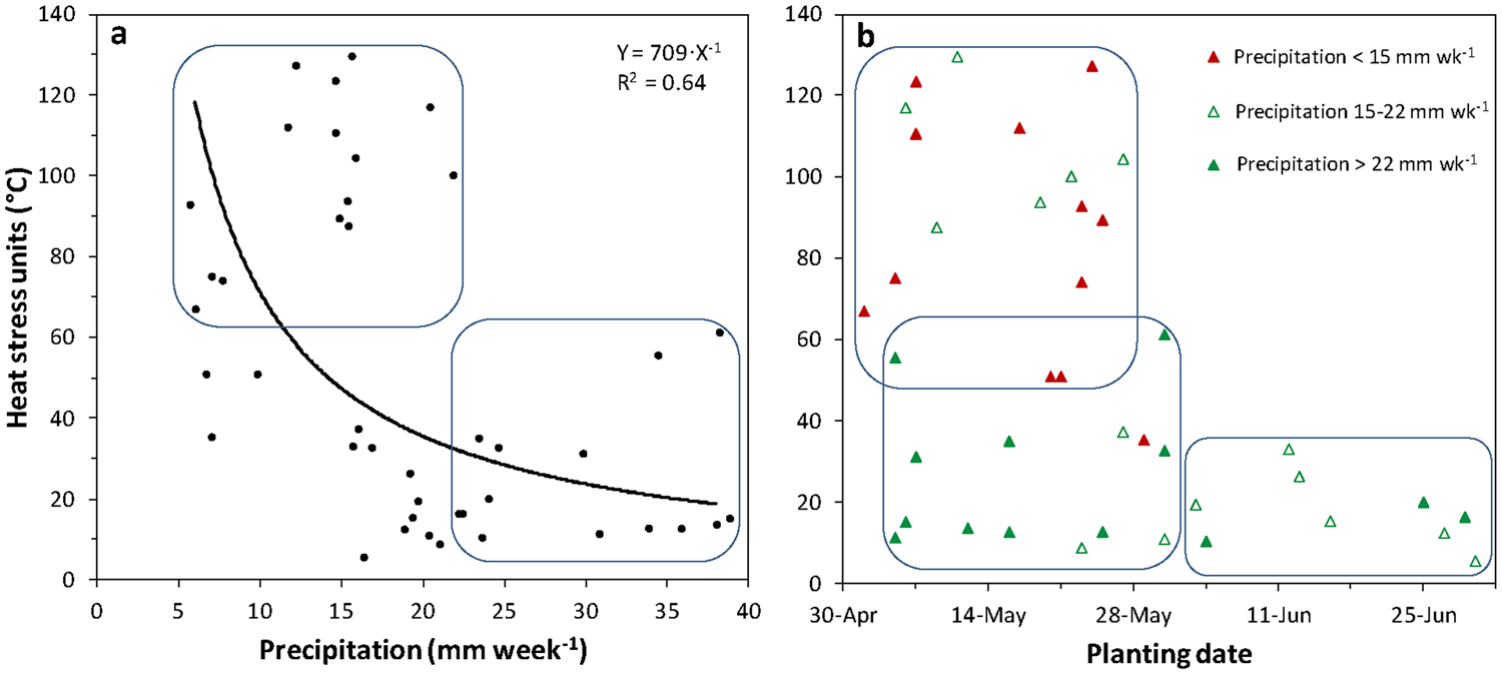
Heat stress units (accumulated daily maximum temperature in excess of 30 °C) as a function of (**a**) late critical period weekly precipitation and (**b**) planting date of corn. The upper box in 1a defines an area with heat units >60 °C and precipitation <22 mm wk^−1^, and the lower box defines an area with heat stress units <60 °C and precipitation >22 mm wk^−1^. The points in 1b are coded by precipitation categories displayed in the legend. Note that the top left box is composed mostly of points with low precipitation (<15 mm wk^−1^), the lower left box of points with high precipitation (>22 mm wk^−1^), and the lower right box of points with intermediate precipitation (15–22 mm wk^−1^).

Heat stress was hyperbolically related to late critical period precipitation for corn, whereby high heat stress was associated with low precipitation and low heat stress was associated with high precipitation (Fig. 1a). These variables are often, but not necessarily, related^4, 5^. This relationship is partially explained by the relation of heat stress with corn planting date. High heat stress was only found when corn was planted in May, but heat stress was low when corn was planted in June (Fig. 1b). When planting was delayed until June, the critical period for corn reproductive development tended to miss the most extreme heat that often occurred in mid-July. It is interesting to note that May-planted corn included both the lowest precipitation events that were associated with high heat stress, and the highest precipitation events that were associated with low heat stress (Fig. 1b). This result provides little guidance to growers in selecting an optimum planting date for avoiding precipitation and heat stress conditions.

Several reports relating county-level corn yields with monthly precipitation in the midwestern US have also shown positive associations between early or mid season precipitation and corn yields and negative associations between mid season temperature and yield^5, 22–24^. Analysis of data from over one-hundred years at a long term experiment in Missouri determined that the optimum weather pattern for corn was less precipitation and warmer temperatures in the planting period, a rapid increase in precipitation and warmer temperatures during emergence, and more precipitation and cooler-than-average temperatures in the anthesis and kernel-filling periods^25^. Corn is more sensitive to drought stress from V12 to denting stage than during other phenological stages^2, 26^, and these stages correspond with the late critical period identified by our data. Drought in the late vegetative period (V12–V16) negatively affects ear formation and tassel emergence, while stress during pollination and fertilization reduces kernel formation and yield^27^. Likewise, high heat stress during this critical period also contributes to drought stress through increasing vapor pressure deficit, which increases demand for soil water to sustain a given rate of carbon assimilation and reduces soil water availability by raising evapo-transpiration rates^4^. Vapor pressure deficit during the third month after planting (during late vegetative and early reproductive periods) was most influential in determining corn and soybean yields in the US Corn Belt^3^.

Weed cover was not significantly correlated with yield in conventional corn and soybeans, and was not a significant factor in multiple regression analyses of these crops (Table 1). In contrast, significant correlation and standardized coefficients for weed cover in organic systems suggested that weed competition had a significant influence on yield in organic systems. Previous research showed that corn yield reduction due to weeds at FSP ranged from 4 to 76%, with the highest yield loss occurring during years with below-average precipitation and the lowest yield loss during years with above-average precipitation^20^. Consequently, weed competition probably contributed to the overall impact of drought on lowering crop yields in organic systems at FSP. Other management variables had no impact on yield (Table 1). Preplant tillage was the principle factor distinguishing the no-tillage and chisel-tillage conventional systems, but this variable had no significant effect on yield in either corn or soybeans. Rotational diversity (as quantified by the number of rotational years without a row crop) was the primary variable distinguishing organic systems, but this variable had no significant effect on yield.

Although nitrogen is known to have a major role in determining corn yield, nitrogen was not included as a variable in these analyses. Both no-tillage and chisel-tillage conventional systems received similar amounts of fertilizer so this factor would not be expected to have explanatory power in differentiating conventional yields. Organic amendments including legume cover crops, alfalfa, and/or poultry litter were applied to give similar available nitrogen each year, so these also would not be expected to differentiate organic yields. Measurements of available soil nitrogen in each plot each year were not available. However, analysis of total and mineralizable soil nitrogen after two rotational cycles demonstrated little difference between no-tillage and chisel-tillage conventional systems or among the three organic rotations^18^.

### Conventional versus organic yield comparison

Because crop yields were highly influenced by inter-annual meteorological conditions, a comparison of crop yields under conventional and organic management required adjustment to remove meteorological influences. Also, because crops were often planted at different times and therefore subjected to different meteorological conditions in the same year, a simple analysis of variance with blocking for each year would not remove all meteorological effects. Consequently, an analysis of covariance was performed with important precipitation and temperature variables as co-variables to adjust yield to common conditions. When adjusted for precipitation and heat stress, there was a significant difference between yield of conventionally and organically managed corn (P < 0.05). The least squared mean of conventional corn yield was 6660 kg ha^−1^ whereas that for organic corn was 4610 kg ha^−1^, representing a 31% reduction with organic management. This yield reduction in organic compared to conventional corn at FSP is higher than average yield reductions that have been reported in recent meta-analyses of organic yields^12, 13^. Despite the higher yield capacity of soils in FSP organic versus conventional management systems after two rotational cycles (attributed to greater soil nitrogen mineralization potential^18^), corn yields have remained consistently lower in organic systems throughout the course of this experiment. Poor weed control leading to weed competition is an important factor influencing organic corn yield. When weed cover was added as a covariate along with precipitation and heat stress in the analysis of covariance model, the yield reduction of organic relative to conventional management was reduced from 31 to 16%. This suggests that poor weed control in organic corn accounted for a significant portion of the yield differential between organic and conventional yield, but did not account for all of it. Reduced crop populations have also been identified as contributing to this yield deficit^16, 28^, while the possibility that organic corn cultivars have lower yield potential than conventional cultivars may be an important consideration that has not been documented. Preliminary investigations at FSP suggest that organic corn cultivars do yield lower than conventional corn cultivars when planted on the same date and grown with the same production practices (Cavigelli, unpublished data).

After adjustment for precipitation and heat stress, the least squared mean of conventional soybean yield was 3040 kg ha^−1^ whereas that for organic soybean was 2420 kg ha^−1^, representing a 20% reduction with organic management. Similar differences between conventional and organic yields have been found in meta-analyses of soybean^12, 13^. When weed abundance was added as a covariate along with precipitation and heat stress in the soybean analysis of covariance model, the yield reduction of organic relative to conventional management was reduced from 20 to 7%. This suggests that poorer weed control in organic soybean accounted for most of the yield difference between management practices. Previous analyses of FSP soybean yield demonstrated a similar conclusion^28^.

### Yield-precipitation efficiency

**Figure 2:**
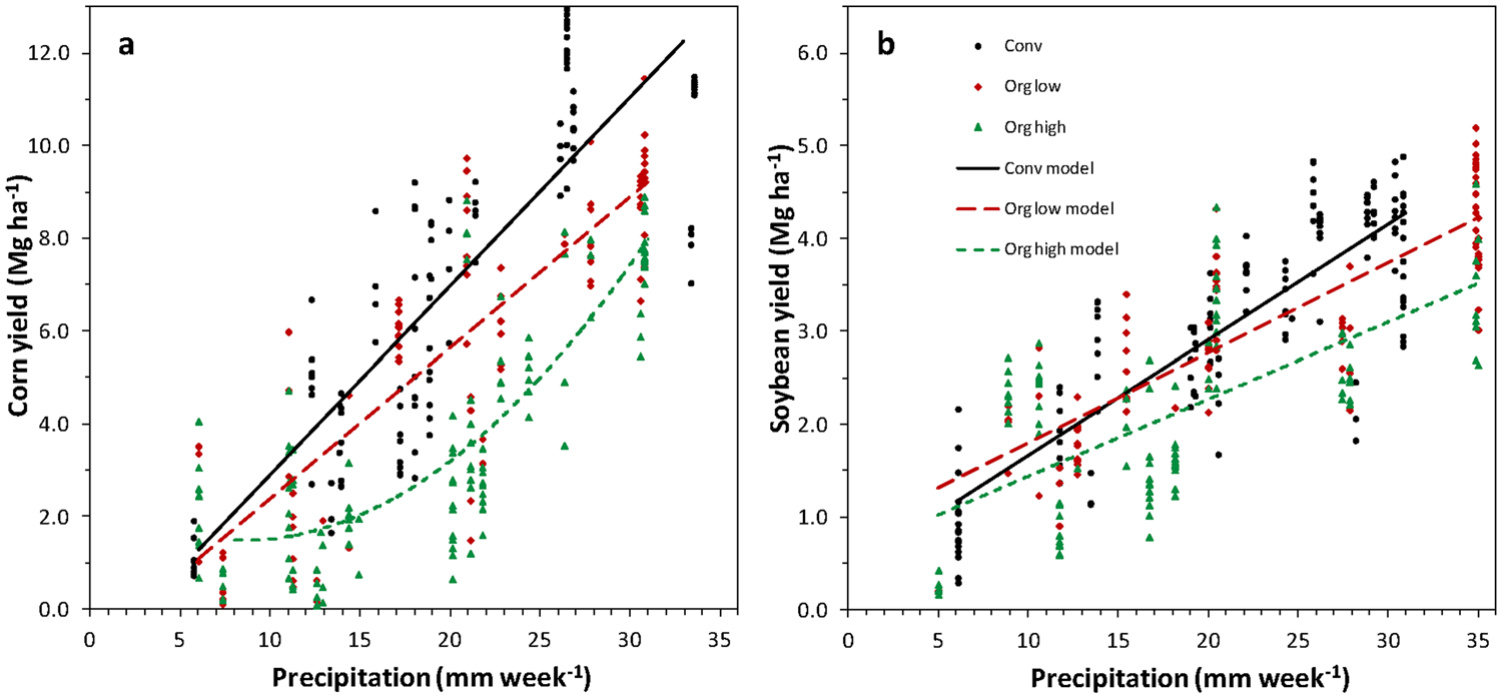
Yield response of (**a**) corn and (**b**) soybean to precipitation during the most beneficial period. Regressions were computed for conventional systems (Conv), organic systems with weed cover <25% (Org low), or organic systems with weed cover ≥ 25% (Org high). Corn models were Y = 0.408X − 1.183 (R^2^ = 0.71, n = 112) for Conv, Y = 0.326X − 0.863 (R^2^ = 0.70, n = 85) for Org low, and Y = 0.0128X^2^ − 0.215X + 2.83 (R^2^ = 0.68, n = 117) for Org high, where Y = yield and X = weekly precipitation. Soybean models were Y = 0.125X + 0.418 (R^2^ = 0.72, n = 133) for Conv, Y = 0.0973X + 0.825 (R^2^ = 0.71, n = 87) for Org low, and Y = 0.0831X + 0.606 (R^2^ = 0.44, n = 104) for Org high. Covariance analyses were performed with first order models, but a second order model was significant (P < 0.0001) for Org high corn and is presented in this graph.

Given the strong influence that precipitation has in determining crop yields as shown in this and many other reports^5, 22–25^, the efficiency of grain production per unit of precipitation for conventional and organic management was explored. In this case, an analysis of covariance was used to compare yield-precipitation slopes for conventional and organic management. This analysis showed significant management by precipitation interactions for all contrasts between conventional and organic management groups (Supplementary Table S3) and significantly higher yield per unit precipitation slopes with conventional than with organic management for both corn and soybean (Fig. 2). This suggests that conventionally-managed crops at FSP used precipitation more efficiently than organic crops. The presence of weeds that compete for soil moisture is clearly responsible, in part, for lower efficiency in organic crops. Higher percent weed cover in the organic high (mean = 53%) than organic low (12%) weed group undoubtedly resulted in lower yield per unit precipitation in the high-weed group, although this difference was only significant for organic corn (Supplementary Table S3). In addition, although the same weed cover threshold defined both the conventional and the low-weed organic groups, the average percent weed cover was lower in the conventional than in the low-weed organic group (4 versus 12% in corn and 2 versus 12% in soybean). So, a portion of the difference between the conventional and low-weed organic groups may still have resulted from greater competition with weeds for soil moisture in the low-weed organic group.

There is currently a global water gap (the difference between current and potential production in the absence of water constraints) of 29% for rain-fed areas and 6% for irrigated areas of the world^29^. Integrated water management strategies that combine irrigation improvements and water conservation practices such as reduced tillage and mulching could close this water gap by up to 62%^29^. Given the critical importance of efficient use of available precipitation for production^27, 30^, reduced-tillage systems could offer more efficient use of water resources for production of organic corn and soybean. Current organic management, including that at the FSP site, is characterized by several tillage events for seedbed preparation and for post-planting control of weeds. Reduced-tillage approaches with high cover crop residue may be an option that could improve the water use efficiency of organic systems, although recent research suggests that soil moisture extraction by cover crops that precede the cash crop and weed control challenges could limit the potential of these systems^31^.

### Yield periodicity and potential El Niño Southern Oscillation associations

**Figure 3:**
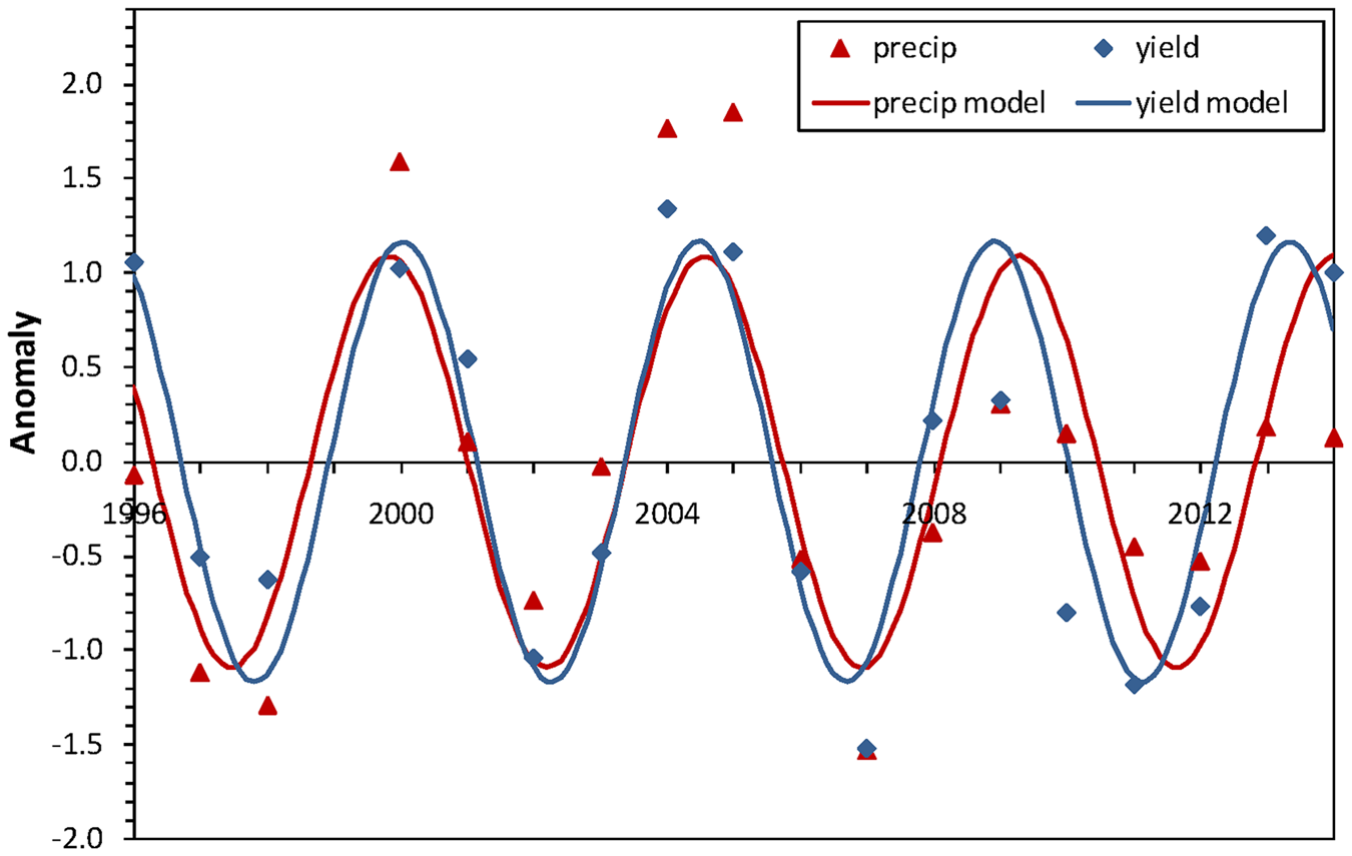
Periodic models of corn yield and late season precipitation anomalies. Points represent average annual anomalies for ease of visualization, but the full data set was used for analysis (n = 325 for yield and n = 328 for precipitation). Model parameter values are presented in Supplementary Table S4 for combined conventional and organic corn data.

Corn yields exhibited surprisingly regular fluctuations between high and low values over the course of this experiment (Supplementary Fig. S1). A model with a cosine-based periodic function fit de-trended, standardized corn yield data well, accounting for 72% of the variability (Supplementary Table S4). Late season precipitation, which was the primary driver of corn yields, exhibited a nearly identical periodicity as that described by the corn yield model (Fig. 3). The period for each cycle of corn yield was 4.4 years, whereas the period for precipitation associated with corn yield was 4.7 years. Because data was de-trended and standardized, the influence of management factors were eliminated and the remaining anomalies represented primarily annual meteorological effects, resulting in almost identical response patterns for corn yield and precipitation (Fig. 3).

The distinctive periodic pattern of corn yield fluctuations observed in this project and its close association with precipitation patterns suggest a possible association with global meteorological patterns that may drive local weather. The approximately four and a half year period defining FSP precipitation and yield fluctuations is similar to the approximately four year period (range 2 to 7 years) for the El Niño Southern Oscillation (ENSO) that is known to have teleconnections to climate anomalies across the world^32, 33^ and in the US^34^. El Niño Southern Oscillation refers to a pattern of alternating equatorial Pacific Ocean surface temperatures and a commensurate alternation of atmospheric pressure between the eastern equatorial Pacific and Indian Ocean^32, 33^. Climate anomalies associated with ENSO events can result in significant global crop yield anomalies^35^. Corn productivity in the midwestern US was shown to be related to ENSO behavior^36^.

**Figure 4:**
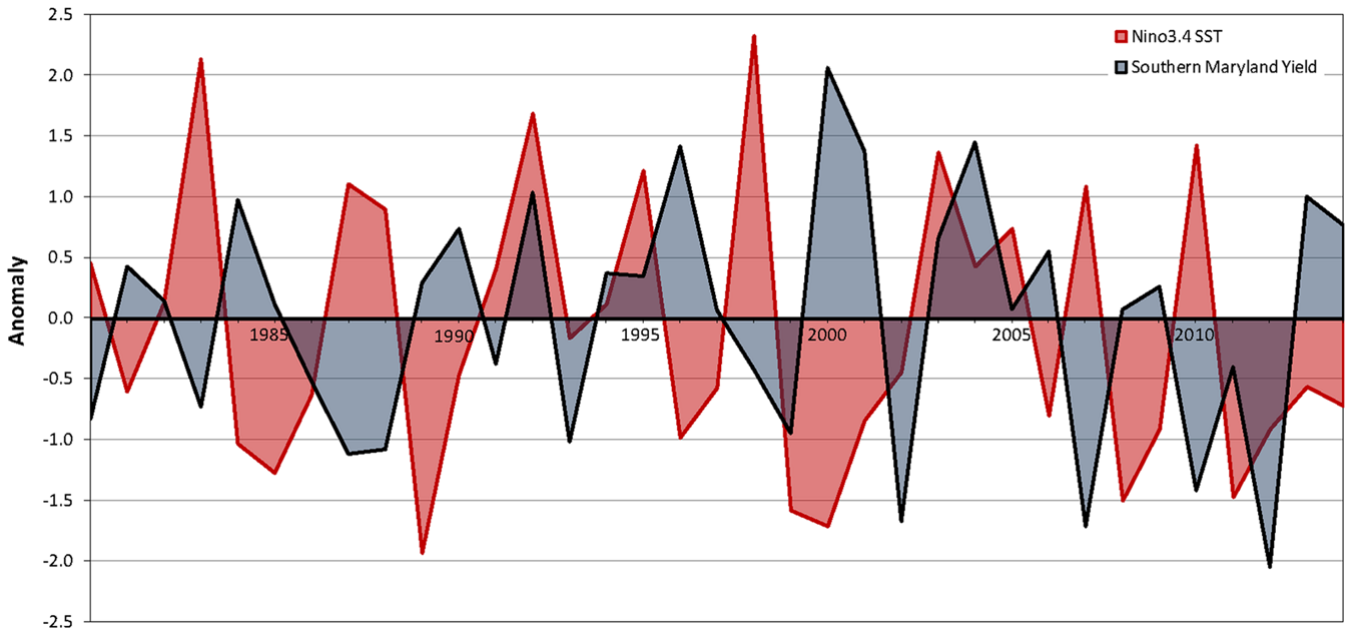
Yield anomalies of conventionally managed corn in Southern Maryland counties and sea surface temperature (SST) anomalies in the Pacific NINO3.4 region from 1980 to 2014. SST anomalies are based on the maximum (or minimum) values during the winter preceding the season for which corn yield was obtained.

The ENSO patterns are measured by sea surface temperatures (SST) in the NINO3.4 region of the Pacific, which fluctuate with maxima (El Niño) or minima (La Niña) typically occurring during the boreal winter (Supplementary Fig. S4)^32^ and transitions occurring during the summer when most agricultural production occurs. A database was developed for examining relationships between NINO3.4 SST winter maxima (or minima) and yield or precipitation in Maryland over a 35 year period. The dataset used Southern Maryland corn yield data and Beltsville, Maryland, precipitation data for 1980 to 2014 so as to provide a longer time frame for making inferences than could be obtained from the 18-year dataset at the FSP site. Corn production in the counties of Southern Maryland was considered to be similar to conventional management at FSP, being primarily rain-fed with similarly shallow, coastal plain soils. Corn yield anomalies for FSP conventional management and Southern Maryland during 1996 to 2014 showed similar fluctuation trends and a high correlation (r = 0.77). Inspection of the relation between Southern Maryland corn yield and SST anomalies from 1980 to 2014 showed some years with an inverse relationship (Fig. 4). Positive SST anomalies (El Niño) were associated with negative yield anomalies in 1983, 1987, 1998, 2007, and 2010; while negative SST anomalies (La Niña) were associated with positive yield anomalies in 1984, 1989, 1996, and 2000. In other years, there was little consistent association, and, consequently, the overall correlation of Southern Maryland corn yield and SST anomalies was low (r = −0.27). Assuming that ENSO teleconnections with local Maryland weather could have been offset by one or more years, SST anomalies for various lag periods were compared with Southern Maryland yield anomalies. However, correlations were low between Southern Maryland yield anomalies and SST anomalies from three winters before (r = 0.19), two winters before (r = 0.03), the winter before (r = −0.27), and one winter after (r = 0.14) the corn growing season (Supplementary Fig. S5).

Precipitation anomalies at FSP during the late critical period for conventional corn in 1996 to 2014 were highly correlated with July precipitation anomalies at Beltsville, Maryland, during this same period (r = 0.85). Visual inspection of Beltsville July precipitation anomalies from 1980 to 2014 compared with SST anomalies showed similar inverse relationships in some years, as were observed with Southern Maryland corn yield anomalies. Positive SST anomalies were associated with negative precipitation anomalies in 1983, 1987, 1998, and 2007; while negative SST anomalies were associated with positive precipitation anomalies in 1984, 1989, 1996, and 2000 (Supplementary Fig. S6). However, considering all years, there were low correlations between July precipitation and SST anomalies that occurred from three winters before to one winter after the precipitation period (correlation coefficients ranged from −0.17 to 0.15, Supplementary Fig. S5). Therefore, the lack of correlation between either Southern Maryland yields or July precipitation and SST anomalies for this 35 year period suggests that observed local patterns are unlikely to have occurred as a direct result of teleconnections to ENSO cycles. There is no question that ENSO activity has a profound influence on global weather patterns^32^, but local weather probably is modulated by a sufficiently large number of factors so as to preclude consistently predictable relationships to ENSO cycles^34^.

Data presented in this paper show that corn and soybean yields at the FSP site in the US mid-Atlantic coastal plain from 1996 to 2014 were largely driven by fluctuations in precipitation and heat stress. Given the demonstrated periodic occurrence of droughts in the mid-Atlantic area, it is recommended that water mitigation strategies become a high priority. Despite a tenuous association between ENSO and crop performance in Maryland, the projected increased frequency of extreme ENSO events may still result in an overall increased frequency of extreme local weather events^37^. Integrated water management including irrigation and water conservation practices can form a reasonable mitigation strategy^29^, but water usage will need to be balanced with urban demand for water in this highly populated region of the US. Organic farming has many environmental and nutritional merits, but lower yields and inferior precipitation use efficiency, as illustrated in this paper, suggest that alternative systems will be needed to grow these crops sustainably in this region. This may involve hybrid systems integrating the best of conventional no-tillage systems with the soil improving aspects of organic rotations^8, 9, 12^. Or it may involve shifting production away from a water and resource intensive crop such as corn. There is advocacy for production of crops that more closely match human nutritional requirements^38^, and this approach may be highly desirable in this densely populated region of the US. Integrated grain and vegetable production rotations need to be developed that utilize all seasons including those with low vapor pressure deficits and that would be more resilient to periodic climatic fluctuations.

## Methods

### Farming System Project (FSP) site description and experimental design

The FSP is a long-term agroecosystem experiment that was initiated in 1996 at the USDA-ARS Beltsville Agricultural Research Center in Beltsville, MD. Primary soil types are Christiana (fine, kaolinitic, mesic Typic Paleudults), Matapeake (fine-silty, mixed, semiactive, mesic Typic Hapludults), Keyport (fine, mixed, semiactive, mesic Aquic Hapludults), and Mattapex (fine-silty, mixed, active, mesic Aquic Hapludults) silt loams. The experiment is designed around five cropping systems: 1) conventional no-tillage, 2) conventional chisel-tillage, 3) organic with a two-year corn (*Zea mays* L.)-soybean [*Glycine max* (L.) Merr.] rotation, 4) organic with a three-year corn-soybean-wheat (*Triticum aestivum* L.) rotation, and 5) organic with a six-year corn-soybean-wheat-alfalfa-alfalfa-alfalfa (*Medicago sativa* L.) rotation. The conventional systems initially had a two-year corn-wheat/double crop soybean rotation, but were expanded in 2001 to include full-season soybean in a three-year corn-soybean-wheat/double crop soybean rotation. Conventional systems were managed with herbicide and fertilizer programs and current genetically modified cultivars according to recommendations by a panel of extension personnel. Organic systems were managed according to USDA National Organic Program standards and recommendations by a panel of organic farmers. Complete experimental and operational details of cropping systems have been published elsewhere^28^. There is no irrigation available at the FSP site, so all systems are representative of rain-fed production.

The experiment was designed as a split-plot design with cropping system assigned to whole plots and crop rotation phase assigned to subplots. Each cropping phase of each rotation was represented every year. Cropping systems were replicated in four randomized complete blocks. Subplots measured 9.1 m wide and 111 m long (0.1 ha in size). The experimental area was planted to no-tillage corn for three seasons before plots were established in 1996, thus, the conventional no-tillage system was considered an extension of preexisting farming practices and data were considered representative of this treatment from 1996 to 2014. The three organic systems were initiated in the spring of 1996 with an herbicide treatment to kill existing vegetation, so rotations using certifiable organic practices were not fully operational until 1997. The 18-year experimental period from 1997 to 2014 represents nine two-year, six three-year, and three six-year rotational cycles. We focus on corn and full-season soybean in this paper since wheat and double crop soybean were not represented in every system.

The middle rows of each corn plot were harvested across the entire plot length by combine. Grain was weighed and yield adjusted to 15.5% moisture content. The middle rows of conventional soybeans (drilled in 19 cm rows) and of organic soybeans (planted in 76 cm rows) were harvested by combine and adjusted to 13.5% moisture content. Yield data were not available for 1999 when extreme drought killed most crops before harvest, for 2003 conventional corn when standard management practices were not performed because of excessively wet early-season weather, and for 2010 organic soybean when seed shattered before harvest.

### Precipitation and temperature critical periods and factors influencing yield

An initial assessment of variance components was conducted to determine the primary factors affecting corn and soybean yield. Variance decomposition was estimated for year, management group (conventional or organic), systems nested within group (no-tillage versus chisel-tillage within conventional, or two-year versus three-year versus six-year rotation within organic), and replicated field block. The restricted maximum likelihood method was used for computing estimates^39^.

Meteorological data were obtained from a station at the FSP site. To determine the influence of weather on yield patterns, critical periods for corn and soybean development were identified. Generally, early season conditions around planting and crop establishment and late season conditions around late vegetative and early reproductive growth have been demonstrated as most critical for determining crop yields^5, 22, 24, 25^. Thus, correlations between yield and climatic parameters covering a range of weeks between 4 weeks before and 4 weeks after planting were explored to identify the critical early period, and correlations between yield and climatic parameters covering a range of weeks between 6 and 15 weeks after planting were explored to identify the critical late period. The weekly range for a given climatic parameter that had the highest correlation with yield was defined as the critical period and was used for analyses described below (critical periods are listed in Supplementary Table S1 and critical period precipitation is presented in Supplementary Fig. S3).

The climatic parameters examined included precipitation per week, average temperature per week, and heat stress unit accumulation. Heat stress units (*HSU*) were computed based on a 30 °C threshold^4^

1 $$HSU={\sum }_{i=1}^{n}(T{\max }_{i}-30\,^\circ C)$$

where *T*max is the daily maximum temperature and the summation was accumulated daily over the critical period of *n* days, but only for days when *T*max exceeded the threshold. This parameter was evaluated only during the late critical period, because there were few days during the early period where *T*max exceeded the threshold. Planting dates for corn and soybean varied considerably from early May to late June depending on weather and operational constraints, so it was important to identify critical periods relative to planting dates in each year rather than according to a specified month or other fixed range of calendar dates. See Supplementary Information for additional details.

An analysis of factors influencing corn or soybean yield was conducted using meteorological and agronomic data. For conventional systems, this analysis included early and late critical period precipitation, early and late critical period average temperature, heat stress units, a preplant tillage dummy variable (0 = no-till, 1 = chisel-till), and weed cover (percentage of soil area covered by weeds at weed maturity). For organic systems, these factors included the same variables identified above (with the exception of tillage) as well as the number of years in the crop rotation without a row crop (0 for two-year rotation, 1 for three-year rotation, and 4 for six-year rotation). All variables were de-trended when they exhibited a significant linear trend across the experimental period. De-trended data was the deviation of the original data from the predicted value of the linear trend. The Pearson correlation among these variables was determined and the multiple regression of these variables on yield was computed using a stepwise selection process with P < 0.01 as the selection criterion for entry and retention in the model^39^. Standardized regression coefficients, which are scaled according to the standard deviation ratio of the dependent and independent variables^39^, were used to assess the relative importance of variables selected for each model.

## Determination of the most beneficial period for precipitation

Given that both early and late season precipitation played important roles in determining yield, we explored combining these variables to give a single precipitation parameter that could accurately predict crop yields in subsequent analyses. The combined value was essentially the weighted average of the early and late critical periods, with weights reflecting the number of weeks present in each period,

2 $$WAP=\frac{{\sum }_{i=1}^{e}p{e}_{i}+{\sum }_{i=1}^{l}p{l}_{i}}{e+l}$$

where *WAP* is the weighted average precipitation, *pe* and *pl* are the weekly precipitation values for the early and late critical periods, and *e* and *l* are the number of weeks in the early and late critical periods, respectively. Computation of regressions between yield and the weighted average precipitation confirmed that R-squared values were higher than for either early or late critical periods separately, with the exception of organic soybean (Supplementary Table S2). Thus, the weighted average critical period was defined as the most beneficial precipitation period for conventional and organic corn and for conventional soybean, but the late critical period was the most beneficial period for organic soybean. The most beneficial precipitation values were used for covariance analyses described below.

### Analyses of conventional and organic yields and yield per unit precipitation efficiency

Comparisons of conventional and organic yields were performed by analysis of covariance in two ways. First, in order to compare conventional and organic yields in the absence of variability associated with climatic factors, an analysis of covariance was conducted with the most beneficial precipitation period and heat stress as regression variables and crop management (conventional or organic) as class variable. The least squared mean provided a comparison of yields adjusted for these meteorological variables^39^. A follow-up analysis of covariance was conducted with weed cover added to the meteorological regression variables in order to assess the relative impact of weed competition on the yield differential between conventional and organic management.

Second, an analysis of covariance was performed to compare the slopes of crop yield per unit precipitation for conventional and organic management. This analysis was conducted with management group as a class variable, precipitation during the most beneficial period as regression variable, and a management group by precipitation interaction term^39^. Because weed competition could potentially influence the efficiency with which crops used available soil moisture, data were segregated into two groups with weed cover < or ≥25%. Since almost all conventional data fell into the low weed group, only data from the low weed group was analyzed for conventional management. For the organic group, this segregation provided a relatively similar number of data points with a similar spread of yield and precipitation values in each weed group; thus, organic management data were split into low-weed and high-weed groups. If the management group by precipitation interaction was significant (P < 0.05) for the three groups, then separate covariance analyses were performed for each management pair. More details on this yield-precipitation analysis of covariance are presented in Supplementary Information.

### Analysis of periodic yield and precipitation patterns

Given the relatively uniform pattern of yield and precipitation fluctuations across years (Supplementary Figs S1 and S3), a periodic trigonometric model was used to define this oscillatory pattern:

3 $$Y=\alpha \,\cos (\rho \pi (T-1996)+\theta )$$

where *Y* = yield or precipitation, *T* = year, *α* = amplitude constant, *ρ* = period constant, and *θ* = an offset angle determining where the function reaches a maximum. The period required for one oscillatory cycle = 2π/*ρ*π = 2/*ρ* years. Data were de-trended and standardized to a mean of 0 and standard deviation of 1 to express data as anomalies in standard deviation units. Because de-trended, standardized data eliminated differences between management practices, conventional and organic data were merged for this analysis (Supplementary Table S4). Models were computed by nonlinear least-squares estimation of parameters that minimized the residual sum of squares^39^. Only corn yield data were used for this analysis because corn data covered a wider range of years than soybean data, and soybean data had a less well-defined periodic structure.

### Yield, precipitation, and sea surface temperature associations

Data on monthly sea surface temperature (SST) anomalies (deviations from a long-term mean) in the equatorial Pacific NINO3.4 region (5°N to 5°S and 120°W to 170°W) were derived from the NOAA National Weather Service Climate Prediction Center^40^. This region defines the standard operational index for ENSO evolution at the Climate Prediction Center^41^. The NINO3.4 SST is characterized by fluctuations with maxima or minima occurring during the boreal winter months (Supplementary Fig. S4)^32^ and transitions occurring during the summer when agricultural production occurs. Since our goal was to investigate associations with annual yield and precipitation values, we simplified the SST dataset to a single annual value corresponding to the maximum (or minimum) value that occurred each winter. A 35-year database from 1980 to 2014 was developed to improve inference relative to the 18-year period comprised by FSP data. Corn yield data from the Southern Maryland district were obtained from the National Agricultural Statistics Service^42^ and precipitation data (recorded at a Beltsville, Maryland weather station located about 2 km from the FSP site) were obtained from the NOAA National Centers for Environmental Information^43^. July precipitation is often identified as highly correlated with yield in long-term yield assessments^5, 22–24^, so Beltsville precipitation data for this month were used as a surrogate for the late critical period precipitation. Data were de-trended relative to the linear trend of the data, and standardized anomalies obtained by normalizing to a mean of 0 and a standard deviation of 1. Anomalies of yield, precipitation, and SST were then assessed for associations by visual observation and correlation analysis.

## Electronic supplementary material

Supplementary Information
