# Supplementary material for: Meteorological fluctuations define long-term crop yield patterns in conventional and organic production systems
Source: Sci Rep. 2017 Apr 6;7:688. doi: 10.1038/s41598-017-00775-8 (PMC5429610; doi:10.1038/s41598-017-00775-8)
Supplement: Supplementary file 1 — Supplementary Information [file 41598_2017_775_MOESM1_ESM.pdf]

Supplementary Information

Meteorological fluctuations define long-term crop yield patterns in conventional and organic production systems

John R. Teasdale[1] & Michel A. Cavigelli[1]

[1] Sustainable Agricultural Systems Laboratory, Agricultural Research Service, United States Department of Agriculture, 10300 Baltimore Avenue, Beltsville, Maryland, 20705. Correspondence and requests for materials should be addressed to J.R.T. (email: john.teasdale@ars.usda.gov)

Crop yields.

Graphic observations confirmed that inter-annual fluctuations represented the highest component of variability in yield data (Supplementary Fig. S1 and S2). There were few and relatively small intra-annual differences between no-till and chisel-till systems within conventional management or between two-year, three-year, and six-year rotations within organic management.

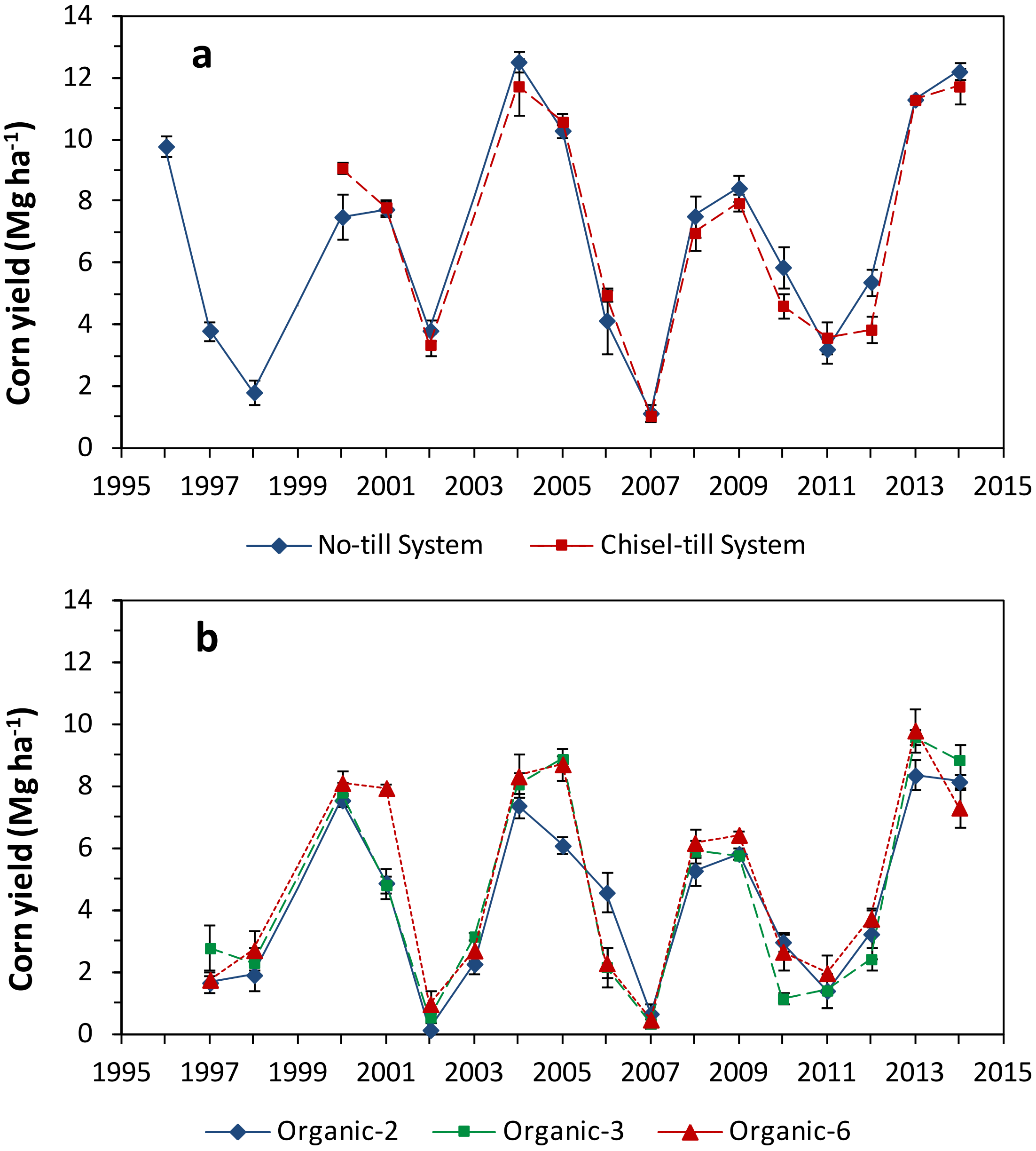

Supplementary Figure S1. Corn grain yield in the (a) conventional no-tillage and chisel-tillage systems, 1996-2014, and the (b) organic systems with 2-year, 3-year, and 6-year crop rotations, 1997-2014. Bars represent the standard error of the mean (n=4).

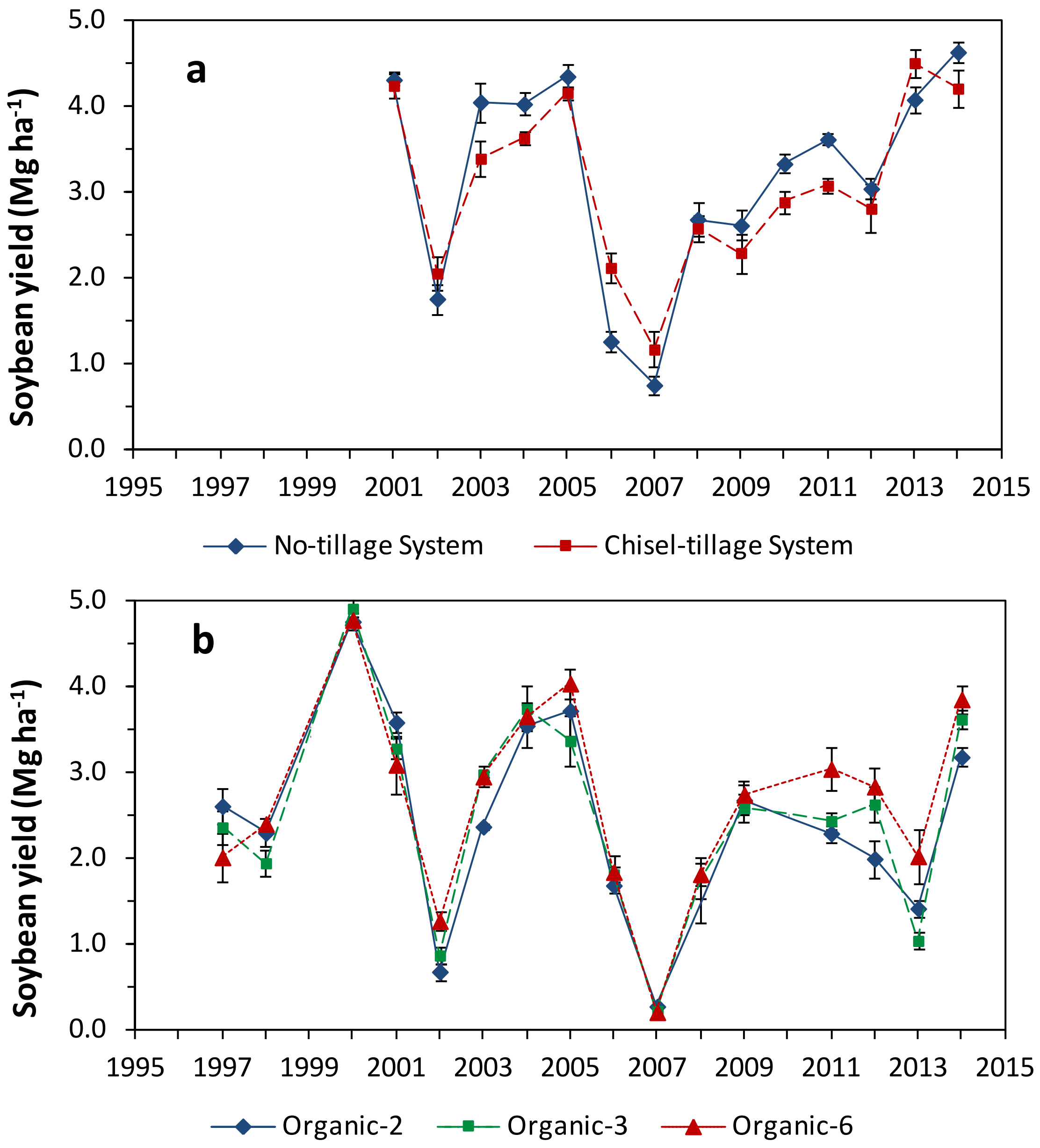

Supplementary Figure S2. Full-season soybean grain yield in the (a) conventional no-tillage and chisel-tillage systems, 2001-2014, and the (b) organic systems with 2-year, 3-year, and 6-year crop rotations, 1997-2014. Bars represent the standard error of the mean (n=4).

Determination of critical periods for precipitation and temperature.

Many reports have analyzed yield in relation to meteorological variables across an extended time period by pairing county average yields and county meteorological data for selected months during the cropping season over a broad region[5,22-24]. This approach can provide general inferences about critical growing-season months for a region, but provides less specificity about the critical timing for optimum precipitation and temperature influences on a crop planted at a specific location and time. Given the availability of our FSP yield dataset at one location over an extended time period, we chose to let our data identify the optimum critical periods for maximizing the impact of climatic variables on yield. Also, given a wide range of planting dates from early May to late June, we computed meteorological variables relative to the planting date for each plot and year.

The early and late critical periods for precipitation and temperature were determined by identifying the range of weeks during crop establishment (early) and reproductive growth (late) that gave the highest correlation between yield and precipitation or temperature. Precipitation and temperature data were compiled for each week before or after planting of each plot in each year and paired with its corresponding yield to obtain correlations over all plots and years. Weekly ranges were explored for their correlation with yield, and the range with the highest correlation with yield was chosen as the critical period for that crop. For example, precipitation during the period from week 9 to 13 had the highest correlation with conventional corn yield (r=0.79), whereas precipitation for week 8 to 13 had a correlation with yield = 0.75, and precipitation during week 9 to 14 had a correlation with yield = 0.68. Thus, the period from week 9 to 13 was designated as the critical period for conventional corn precipitation in later analyses. Critical periods for each variable are shown in Supplementary Table S1.

	Critical weekly period (weeks relative to planting date)			
	Corn		Soybean	
Variable	Conventional	Organic	Conventional	Organic
Early precipitation	-2 to 4	-1 to 2	-2 to 3	-1 to 2
Late precipitation	9 to 13	6 to 11	8 to 13	6 to 12
Early temperature	1 to 3	-2 to -1	1 to 3	-2 to -1
Late temperature	7 to 15	8 to 12	7 to 14	7 to 15
Heat stress units	8 to 13	8 to 13	8 to 13	8 to 13

Supplementary Table S1. Critical weekly periods for precipitation and temperature variables that optimized correlations with corn and soybean grain yields under conventional and organic management. Negative weekly values indicate weeks before planting and positive values indicate weeks after planting. Precipitation variable was total precipitation per week. Temperature variables were average daily temperature and accumulated daily heat units over 30°C.

Corn and soybean under both management systems benefitted from an early critical precipitation period that spanned a week or two before planting to two to four weeks after planting. Optimum moisture during this period would ensure crop establishment, root development, and, in corn, the proper development of leaves, tassels and ear shoots, which were initiated at this time[27,30]. The late critical precipitation period included reproduction and early grain fill in conventional crops[26], but also included late vegetative growth in organic crops. This earlier critical period in organic crops may be explained by the relative advantage crops gained in competing with weeds under abundant soil moisture conditions[20]. Since weeds were controlled in conventional crops, this early advantage was probably less important for optimizing conventional yield.

The early temperature critical period occurred just before planting organic crops, but just after planting conventional crops. This difference may be explained by stimulation of both weed emergence and nutrient mineralization by warm conditions before planting. Emerged weeds would be killed by seedbed preparation and mineralized nutrients would be available for early crop growth of organic crops. These factors would be less important to conventional crops which would probably have benefitted more from warm conditions just following planting to facilitate crop establishment. Finally, all crops under both management systems benefitted from an extended period of cooler weather and low heat stress during the late critical period encompassing late vegetative and reproductive growth.

Precipitation during early and late critical periods followed a relatively similar pattern in a given year for both crops and management systems (Supplementary Figure S3). This is surprising given that corn and soybean were often planted on different dates and organic crops were usually planted later than conventional crops. Despite the disparities in the beginning of critical periods (7 to 58 days for the early critical period and 4 to 27 days for the late critical period), the overall precipitation during these critical periods was relatively similar compared to the inter-annual differences in precipitation.

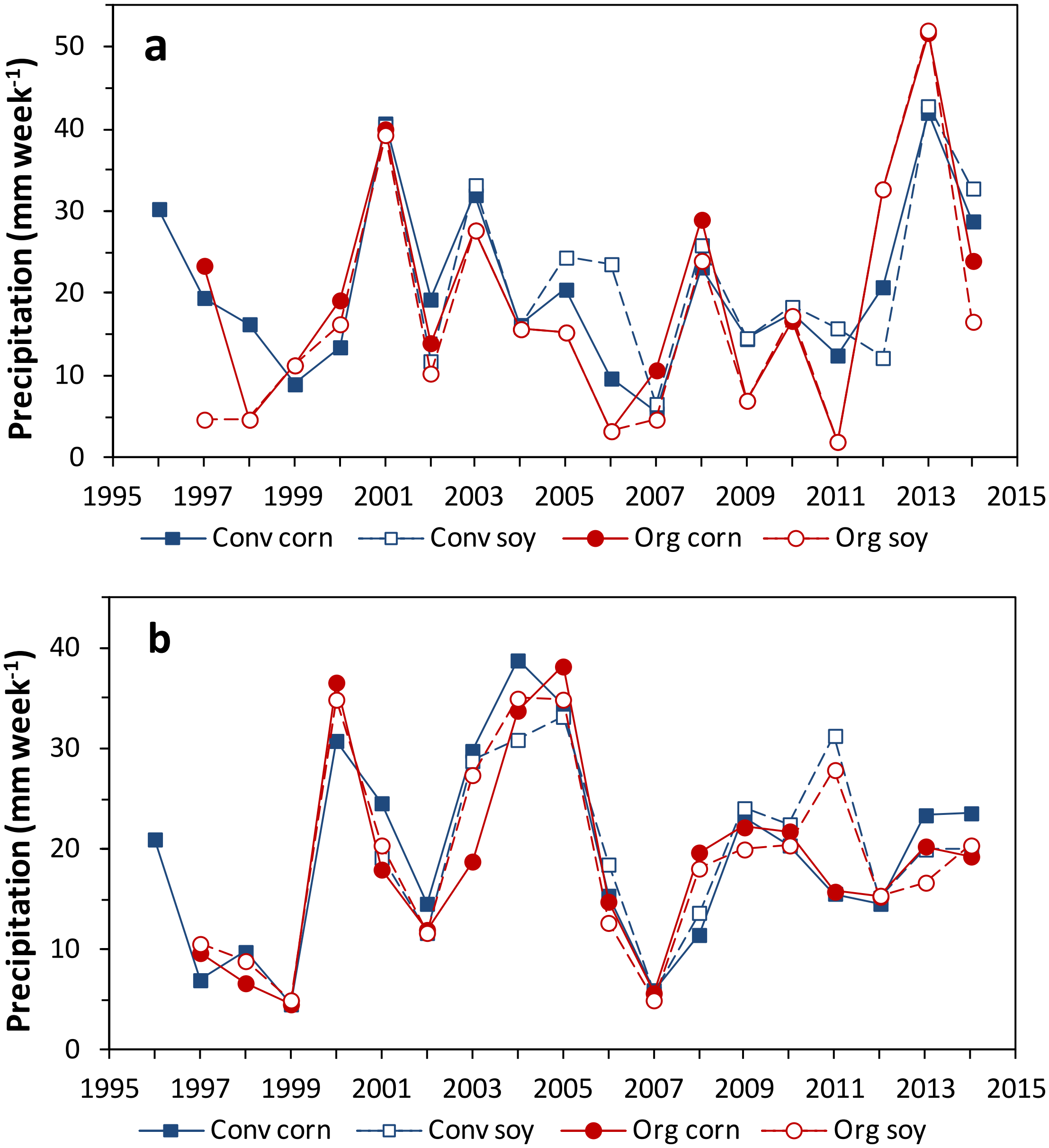

Supplementary Figure S3. Annual precipitation patterns during (a) early season and (b) late season critical periods for conventional corn (Conv corn), conventional soybean (Conv soy), organic corn (Org corn), and organic soybean (Org soy). Early and late season critical periods are defined in Supplementary Table S1.

Determination of the most beneficial period for precipitation.

Regressions between yield and precipitation for the early critical period, the late critical period, and the weighted average of early and late critical periods were determined. Results confirmed that R-squared values were higher for the weighted average period than for either early or late critical periods separately, with the exception of organic soybean (Supplementary Table S2). The weighted average period was defined as the most beneficial precipitation period for conventional and organic corn and for conventional soybean, but the late critical period was the most beneficial period for organic soybean. These most beneficial precipitation values were used for covariance analyses to compare conventional and organic yields and to determine yield-precipitation efficiency as described in the main text.

Crop	Management	n	Early Critical Period	Late Critical Period	Combined Early and Late
Corn	Conventional	123	0.29	0.62	**0.66**
	Organic	202	0.19	0.49	**0.62**
Soybean	Conventional	134	0.47	0.48	**0.71**
	Organic	191	0.01	**0.60**	0.43

Supplementary Table S2. R-squared values for the regression of yield as a function of precipitation for early and late critical periods separately, and of the weighted average of early and late critical periods combined. Values identifying the most beneficial precipitation period for each crop-management combination are shown in bold font.

Yield-precipitation analysis of covariance.

An analysis of covariance was performed for corn and soybean yield, with three management groups (conventional, organic with weed cover < 25%, and organic with weed cover ≥ 25%) as a class variable and precipitation during the most beneficial period as regression variable[39]. A regression analysis of yield versus precipitation was conducted separately for all management groups and found to have a significant first order response, except the organic high-weed cover corn group had a second order response. Consequently, the analyses of covariance used a first order regression model for precipitation. The first covariance analysis contained the three management groups, conventional, low-weed organic, and high-weed organic, and was coded with management, precipitation, and management by precipitation terms in the model. If the management by precipitation interaction was significant (P<0.05), then further covariance analyses were performed for each pair of management, conventional and organic low, conventional and organic high, and organic low and organic high. Results of management group by precipitation interactions are shown in Supplementary Table S3.

Crop	Management groups in model	Probability of a greater F value for model interaction term
Corn	Conv, Org low, Org high	<0.0001
	Conv, Org low	0.0187
	Conv, Org high	<0.0001
	Org low, Org high	0.0300
Soybean	Conv, Org low, Org high	0.0005
	Conv, Org low	0.0050
	Conv, Org high	0.0003
	Org low, Org high	0.2228

Supplementary Table S3. Probability of a greater F value for the management group by precipitation interaction term in covariance analyses of yield by precipitation. Conv = conventional management, Org low = organic management with weed cover < 25%, Org high = organic management with weed cover ≥ 25%.

Periodic model parameters for conventional and organic corn.

The periodic model was fit to conventional corn and organic corn data separately, and both conventional and organic data sets combined. Model parameters were very similar for both conventional and organic data when analyzed separately and when combined. This was expected for de-trended, standardized data where management effects would be removed by this data processing, and where similar precipitation patterns were driving the annual anomalies in both data sets.

Variable	Model specifications	Conventional data	Organic data	Combined data
Yield	Amplitude (α)	1.17	1.18	1.17
	Period constant (ρ)	0.446	0.453	0.451
	Offset angle (θ)	0.471	0.362	0.404
	n	123	202	325
	Model SS/total SS	0.748	0.714	0.724
	Period=2/ρ (years)	4.48	4.41	4.44
Late season Precipitation	Amplitude (α)	1.10	1.08	1.09
	Period constant (ρ)	0.421	0.426	0.423
	Offset angle (θ)	0.949	0.841	0.899
	n	124	204	328
	Model SS/total SS	0.660	0.638	0.646
	Period=2/ρ (years)	4.76	4.70	4.73

Supplementary Table S4. Parameters for the periodic model $Y = \alpha \cdot \cos(\rho\pi(\text{year-1996})+\theta)$ and other model specifications. Corn yield data and late season precipitation data were expressed as anomalies by de-trending and standardizing to mean=0 and standard deviation=1. Model fit was assessed by the fraction of sum of squares (SS) explained by the model relative to the total sum of squares.

Graphs depicting NINO3.4 sea surface temperature and associations with Maryland yield and precipitation datasets.

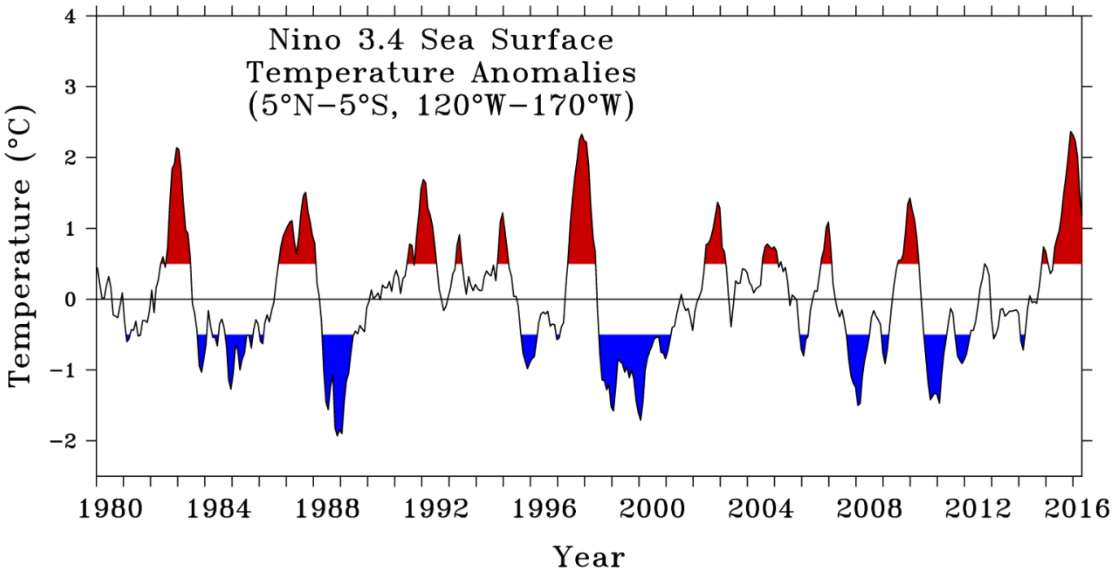

Supplementary Figure S4. Pacific Ocean NINO3.4 sea surface temperature (SST) anomalies. El Niño is an SST period that exceeds an anomaly of 0.5 (identified in red). La Niña is an SST period that is less than an anomaly of -0.5 (identified in blue). Figure courtesy of M. J. McPhaden, NOAA Pacific Marine Environmental Laboratory, Seattle, WA, USA.

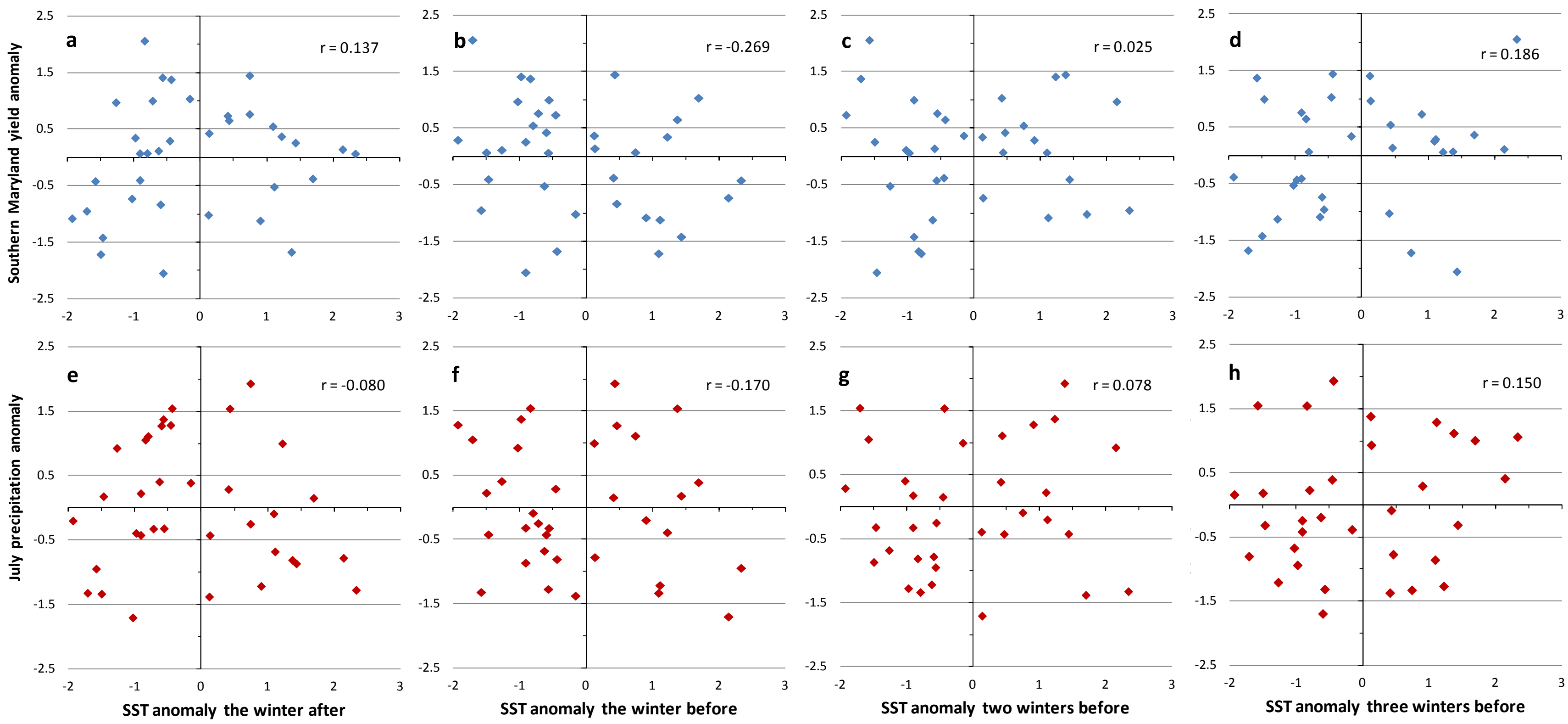

Supplementary Figure S5. Scatter plots of Southern Maryland corn yield anomaly and NINO3.4 SST anomaly (points in blue) during (a) the winter after, (b) the winter before, (c) two winters before, and (d) three winters before the season yield was determined for 1980 to 2014. Scatter plot of Beltsville July precipitation anomaly and NINO3.4 SST anomaly (points in red) during (e) the winter after, (f) the winter before, (g) two winters before, and (h) three winters before the July precipitation for 1980 to 2014. Correlation coefficients are displayed with each graph.

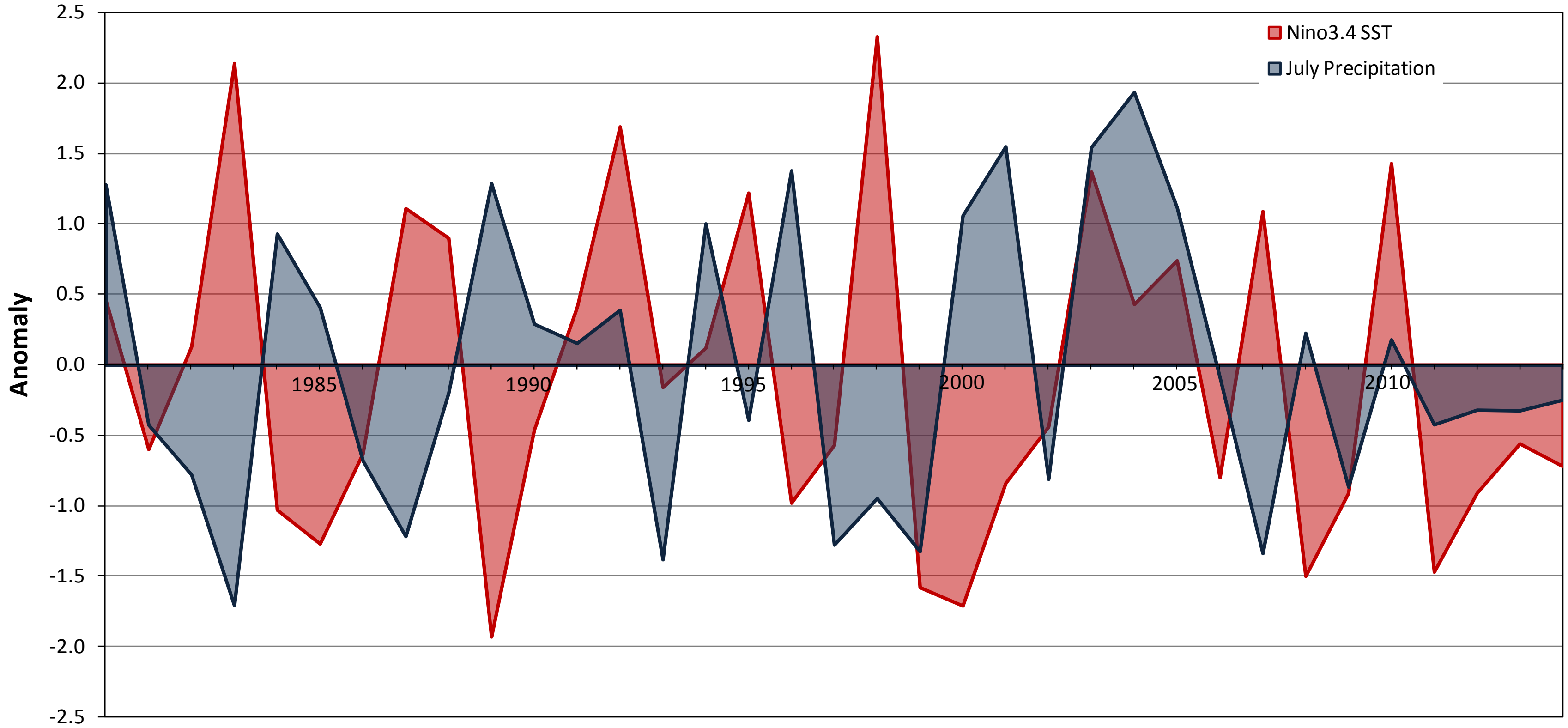

Supplementary Figure S6. NINO3.4 sea surface temperature (SST) anomalies and July precipitation anomalies at Beltsville, Maryland, from 1980 to 2014. Values of SST anomalies were derived from the maximum (or minimum) values during the winter preceding the season for which precipitation was obtained.
